# Association of Plasma Myeloperoxidase with Inflammation and Diabetic status in HFpEF

**DOI:** 10.31083/j.rcm2402056

**Published:** 2023-02-08

**Authors:** Sibille Lejeune, Audrey Ginion, Nassiba Menghoum, David Vancraeynest, Agnes Pasquet, Bernhard L. Gerber, Sandrine Horman, Christophe Beauloye, Anne-Catherine Pouleur

**Affiliations:** ^1^Division of Cardiology, Department of Cardiovascular Diseases, Cliniques Universitaires St. Luc and Pôle de Recherche Cardiovasculaire (CARD), Institut de Recherche Expérimentale et Clinique (IREC), Université Catholique de Louvain, 1200 Brussels, Belgium

**Keywords:** heart failure with preserved ejection fraction, myeloperoxidase, oxidative stress, inflammation, diabetes, vascular stiffness

## Abstract

**Background:**

Inflammation and oxidative stress are thought to play an important role in the pathophysiology of heart failure with preserved ejection fraction (HFpEF) through the development of endothelial dysfunction. Myeloperoxidase (MPO) functions as a link between oxidative stress and inflammation and is an interesting therapeutic target. The objective of this observational cohort study was to compare MPO levels between HFpEF and old controls, to define clinical characteristics associated with high levels of MPO and to assess the relation between MPO levels and vascular function.

**Methods:**

Patients with HFpEF (N = 55) and controls >60 years (N = 18) were prospectively included. All subjects underwent complete echocardiography and blood sampling. MPO levels were dosed by ELISA assay. Effective arterial elastance (Ea) and peripheral arterial tonometry (EndoPAT reactive hyperemia index RHI and augmentation index AIx) were used to assess vascular function. Characteristics between groups defined by the median of MPO were compared using independent samples *t*-test or chi square test.

**Results:**

Patients with HFpEF (80 ± 8.7 years, 65% female) had higher levels of MPO compared to controls (75 ± 5.0 years, 72% female) (34.7 ng/mL [22.7; 44.0] vs 22.6 [18.2; 32.0], *p* = 0.026). MPO levels were correlated with markers of inflammation; C-reactive protein (Pearson’s R = 0.46, *p* = 0.001) and neutrophile to lymphocyte ratio (R = 0.36, *p* = 0.031) and with signs of left ventricular (LV) remodelling and elevated filling pressures, namely NT-proBNP levels (R = 0.32, *p* = 0.019), decreased LV ejection fraction (LVEF, R = –0.36, *p* = 0.008) and E/e’ ratio (R = 0.35, *p* = 0.011). HFpEF patients with levels of MPO above the median were more often men (48% vs 21%, *p* = 0.037) and suffered more often from diabetes (48% vs 18%, *p* = 0.017). Intriguingly, they had lower indices of vascular stiffness (augmentation index 11.1 [0.1; 30.7] vs 19.9 [10.5; 33.4], *p* = 0.018 and arterial elastance Ea 2.06 ± 0.676 vs 2.43 ± 0.721, *p* = 0.065) and there was no difference in endothelial function (1.82 [1.34; 2.30] vs 1.66 [1.32; 1.95], *p* = 0.55).

**Conclusions:**

HFpEF patients have higher levels of MPO than controls, reflecting leukocyte activation and oxidative stress. Among patients, high levels of MPO are associated with male sex, diabetic status, subtle left ventricular dysfunction and pronounced diastolic dysfunction. The association between oxidative stress and vascular stiffness, on the other hand could not be demonstrated.

**Clinical Trial Registration:**

Clinical trial NCT03197350.

## 1. Introduction

Heart failure with preserved ejection fraction (HFpEF) is characterized by signs and symptoms of heart failure, including peripheral oedema, dyspnea and exercise intolerance, in the absence of a reduced left ventricular ejection fraction (LVEF ≥50%) [1]. Current understanding of molecular mechanisms underlying HFpEF is largely based on the 2013 “Paulus and Tschope paradigm” [2] relating coexisting comorbidities to myocardial remodelling and dysfunction, through a systemic pro inflammatory state. Non-cardiac co-morbidities such as diabetes, obesity, hypertension, and chronic kidney disease are common in HFpEF and have the ability to induce systemic inflammation. During inflammation, microvascular endothelial cells produce reactive oxygen species (ROS), which limits nitric oxide (NO) bioavailability leading to oxidative stress and endothelial dysfunction.

Oxidative stress and inflammation are closely interconnected. Transcription factors that regulate the expression of pro inflammatory cytokines are activated under oxidative stress conditions and in turn, induce the generation of ROS, thus creating a vicious cycle of oxidation and inflammation [3]. Myeloperoxidase (MPO), a leukocyte-derived enzyme, functions as a link between oxidative stress and inflammation. During inflammation, MPO is released and uses $H_{2}$$O_{2}$ as a substrate to produce hypochlorous acid, a powerful pro-oxidant and pro inflammatory molecule.

Studies suggest that plasma MPO levels are elevated in patients with HF compared to controls and that increasing levels of MPO are associated with restrictive diastolic stage, right ventricular systolic dysfunction and tricuspid regurgitation in HFrEF [4]. Furthermore, MPO may be involved in the pathophysiology of atrial fibrillation through atrial accumulation of MPO and consequent increase in fibrosis [5]. These results imply that MPO may be important also for the development of HFpEF where diastolic dysfunction, atrial fibrillation and fibrosis are major components. Indeed, a recent study showed that HFpEF patients displayed higher plasma concentration of MPO compared to healthy controls [6]. Furthermore, since oxidative stress and microvascular endothelial dysfunction are suggested as fundamental parts of the pathophysiology and development of HFpEF, MPO inhibition appears as an interesting therapeutic approach and a clinical trial investigating MPO inhibitor “AZD4831” (ENDEAVOR NCT04986202 and NCT03611153) is currently ongoing. Heterogeneity among patients with HFpEF has been singled out to explain the difficulty to find treatments improving prognosis in this population. Hence, identifying characteristics associated with high levels of MPO could be interesting to target subgroups of patients most likely to benefit from treatment with MPO inhibitors.

In this context, the objective of our study was to reinforce data about MPO elevation in HFpEF, to assess the relation between MPO levels and clinical parameters including vascular function and to determine patient characteristics associated with high levels of MPO.

## 2. Methods 

### 2.1 Population

Patients with HFpEF encountered in our division of cardiology between May 2019 and May 2021 were prospectively screened for inclusion in the study. HFpEF was diagnosed according to the guidelines of the European society of cardiology [7, 8]. Briefly, patients had to be symptomatic (New York Heart Association (NYHA) functional class ≥II or hospitalization for HF in the previous 12 months), have a left ventricular (LV) ejection fraction ≥50%, show echocardiographic signs of elevated filling pressures (LV hypertrophy, left atrial (LA) enlargement, elevated E/e’ ratio or elevated pulmonary pressures) and elevated NT-proBNP (>220 pg/mL in sinus rhythm, >660 pg/mL in atrial fibrillation (AF)). The exclusion criteria were: history of reduced ejection fraction (LVEF <50%), severe valvular disease, infiltrative or hypertrophic cardiomyopathy, acute coronary syndrome in the previous 30 days, severe chronic obstructive pulmonary disease, congenital heart disease, pericardial disease, AF with a ventricular response >140 bpm, and severe anemia (hemoglobin <8 g/dL). A total of 55 patients satisfied the inclusion criteria. Patients underwent blood sampling and complete transthoracic echocardiography. All except 10 also underwent endothelial function measurement by endoPAT (6 patients had finger deformities or injuries preventing the probes use and, there was a technical problem with the device on the day of the study for 4 patients). To constitute a control group of similar age and sex, asymptomatic volunteers aged between 60 and 90 years were screened by advertisement in the local community. They all underwent a full clinical exam, blood sampling, ECG, echocardiography and endoPAT. Exclusion criteria were any evidence of heart disease as indicated by clinical history, physical exam and echocardiography. Eighteen subjects satisfied the inclusion criteria. Regarding comorbidities, subjects were considered to have hypercholesterolemia if recorded as pre-existing cardiovascular risk factor in their medical file, if they had elevated cholesterol values or if treated by statins. Diabetes was defined as the presence of a previous diagnosis in the medical record.

The local ethics committee approved the study, and all subjects gave written informed consent before study enrolment (Clinical trial NCT03197350). The investigation conforms to the principles outlined in Declaration of Helsinki. Patients and controls were interrogated about symptoms, medical history and treatment and were thoroughly examined. Other information was retrieved from medical files and from review of hospital records.

### 2.2 Echocardiography

Standardized complete transthoracic echocardiography (TTE) exams were acquired according to established guidelines [9] using iE33 ultrasound systems (Philips Medical Systems, Andover, Massachusetts) equipped with a 3.5/1.75-MHz phased-array transducer and stored on a XCELERA 2.1 PACS server (Philips Medical Systems, Andover, Massachusetts). Annular e’ velocity, average E/e’ ratio, LA volume index and peak TR velocities were measured to evaluate LV diastolic function [10].

### 2.3 Blood Sampling

Blood samples were collected from the cubital vein. Samples were immediately centrifuged and aliquots of plasma and serum were stored in microcentrifuge tubes at –80 ℃ until analysis. Plasma MPO concentration was determined by an enzyme-linked immunosorbent assay (ELISA) method (#DMYE00B, R&D Systems) according to the manufacturer’s instructions.

### 2.4 Vascular Function: Effective Arterial Elastance, Reactive Hyperemia Index and Augmentation Index

**Fig. 1.**
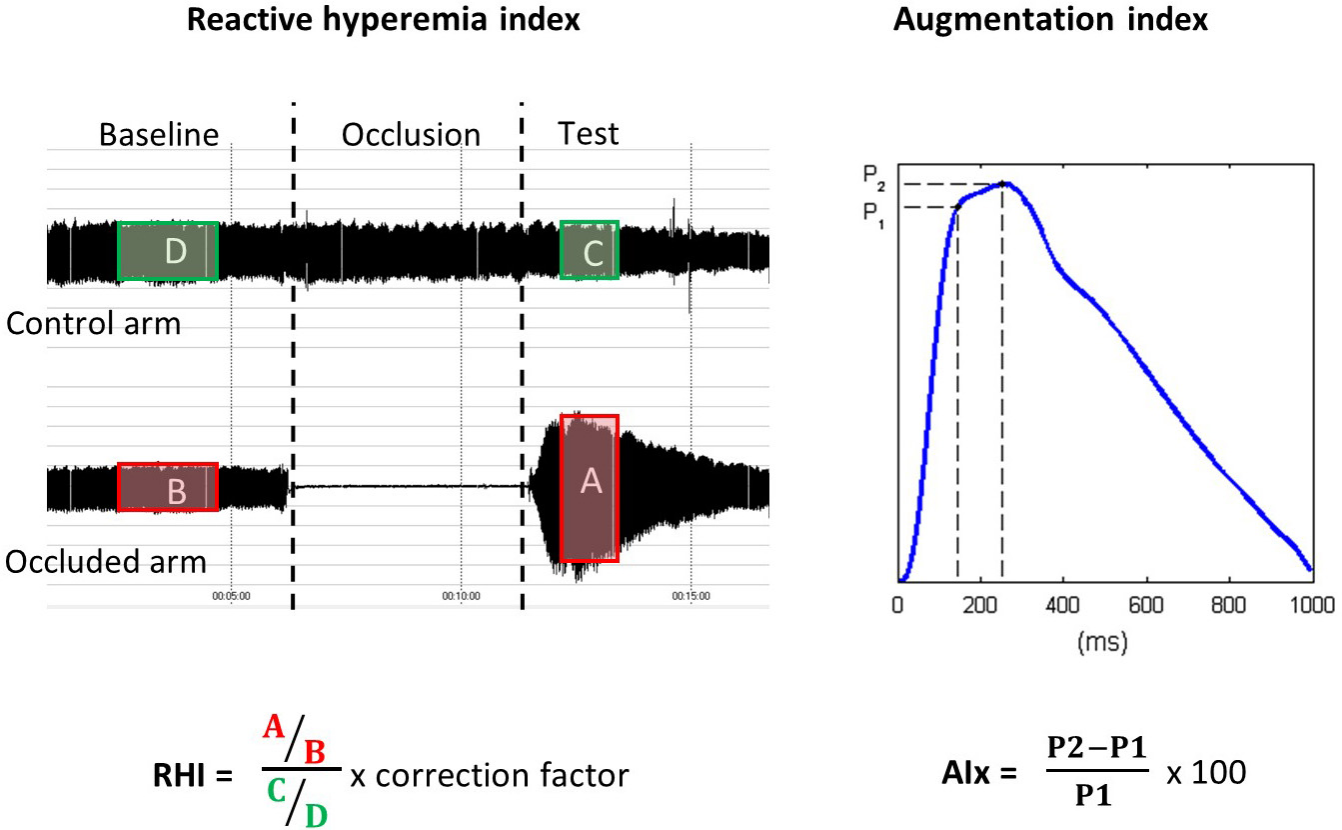
**Calculation of the reactive hyperemia index (RHI) and the augmentation index (Aix) by the EndoPAT2000 software (Itamar Medical)**.

Effective arterial elastance (Ea) was calculated as described in the literature [11]: end-systolic pressure divided by stroke volume. End-systolic pressure was estimated as systolic pressure times 0.9, as previously validated [12]. Digital hyperemia response was measured at finger (index) tips using an EndoPat2000 device (Rev 3, Itamar Medical, Caesara, Israël) (Fig. 1). Briefly, pulse wave amplitude (PWA) changes were assessed as beat-to-beat plethysmographic signals in the index finger by high-sensitive pneumatic probes (EndoPAT, Itamar). The signals were measured at basal state during 5 minutes from each fingertip. Then brachial blood flow was interrupted for 5 minutes by inflation of a sphyngomanometer cuff placed on one proximal forearm, and signals were recorded during occlusion (5 minutes) and after restoration of blood flow (5 minutes). Data were digitized and computed automatically by EndoPat2000 software; the reactive hyperemia index (RHI) was defined as the ratio of mean post-deflation signal (in the 90 to 120-second post-deflation interval) to baseline signal in hyperemic finger normalized by the same ratio in the contra-lateral finger and multiplied by a baseline correction factor (K = 0.523976 × log (mean baseline amplitude) – 0.2). Arterial stiffness was approximated by the augmentation index (AI), which is calculated through software identification of the systolic peak (P1) and reflected wave (P2) inflection points and then using the formula AI = (P1 - P2)/P1 × 100, averaged over multiple valid pulses collected during the baseline period. It is then normalized to heart rate of 75 bpm (referred to as AIx in the manuscript). Lower AI values (including negative results) reflect better arterial elasticity. This method has been shown to correlate well with other methods of AI derivation [13].

### 2.5 Statistical Analysis

Statistical analyses were performed using SPSS version 25 (SPSS Corp., Somers, NY, USA). All tests were 2-sided and *p*-value < 0.05 was considered statistically significant. The sample size of the control group was determined to reach a power of 80%, α = 0.05, with an expected difference of 15% of plasma MPO levels between patients and controls [6] (minimum n = 15). Continuous variables are expressed as mean ± standard deviation (SD) or median [P25; P75] if not normally distributed. Non-normal biomarkers (NT-proBNP, Troponin, CRP, MPO) were log-transformed to achieve normality. Categorical variables are expressed as count and proportion. Receiver operating characteristic (ROC) curves were established and the area under the ROC curves (AUC) were calculated to establish the diagnostic value of MPO levels compared to NT-proBNP levels. Correlation between variables was assessed using Pearson coefficient of correlation (R). Differences of characteristics between groups were examined using independent sample *t*-test, Mann Whitney U test, Chi-square test or Fisher exact test when appropriate. Multivariate logistic regression was used to evaluate the association between MPO levels and diabetic status after correction for age and sex.

## 3. Results 

### 3.1 MPO Levels in HFpEF Compared to Controls 

The characteristics of all 55 patients with HFpEF are presented in Table 1. Patients were 80 ± 8.7 years old, mostly women (65%) and about one third was suffering from advanced heart failure (36% NYHA class III or IV). One third (33%) of the patients had diabetes. While ejection fraction was preserved, patients displayed functional and morphological signs of diastolic dysfunction including increased E/e’ ratio and dilated left atrium. The 18 healthy controls (75 ± 5.0 years) were 72% women (**Supplementary Table 1**).

**Table 1.** **Characteristics of HFpEF patients stratified by levels of myeloperoxidase**.

		All patients (N = 55)	MPO below median (N = 28)	MPO above median (N = 27)	*p*-value
Age (years)		80 ± 8.7	79 ± 9.7	80 ± 7.9	0.72
Female (n, %)		36 (65%)	22 (79%)	14 (52%)	0.037
Body mass index (kg/$m^{2}$)		28.2 ± 4.97	28.3 ± 5.94	28.3 ± 3.84	0.99
Systolic blood pressure (mmHg)		134 ± 20	138 ± 17	129 ± 22	0.099
Diastolic blood pressure (mmHg)		74 ± 14	75 ± 14	72 ± 15	0.45
Heart rate at inclusion (bpm)		72 ± 13	74 ± 12	70 ± 13	0.26
NYHA III – IV (n, %)		20 (36%)	10 (36%)	10 (37%)	0.92
Diabetes (n, %)		18 (33%)	5 (18%)	13 (48%)	0.017
	HbA1C (in diabetic patients) (%)	6.7 [6.35; 8.23]	7.8 [6.40; 9.90]	6.6 [6.15; 7.85]	0.34
Atrial fibrillation (n, %)		42 (76%)	23 (85%)	19 (68%)	0.13
	Paroxysmal (n, %)	10 (18%)	6 (21%)	6 (22%)	
	Permanent (n, %)	32 (58%)	17 (61%)	13 (48%)	
Ischemic cardiomyopathy (n, %)		21 (38%)	8 (29%)	13 (48%)	0.14
Smoking (n, %)		18 (18%)	9 (32%)	9 (33%)	0.93
Hypertension (n, %)		52 (95%)	26 (93%)	26 (93%)	1
Hypercholesterolemia (n, %)		39 (71%)	17 (61%)	22 (81%)	0.09
Sleep apneas (n, %)		6 (11%)	3 (11%)	3 (11%)	1
COPD (n, %)		6 (11%)	4 (14%)	2 (7%)	0.67
Medication					
	Loopdiuretics (n, %)	42 (76%)	19 (68%)	23 (85%)	0.13
	MRA (n, %)	18 (33%)	11 (39%)	7 (26%)	0.19
	Beta blockers (n, %)	34 (62%)	21 (75%)	22 (81%)	0.56
	ACE inhibitors/ARB (n, %)	43 (78%)	16 (57%)	18 (67%)	0.47
	Statins (n, %)	35 (64%)	15 (54%)	20 (74%)	0.11
Biology					
	eGFR (mL/min/1.73 $m^{2}$)	49.4 ± 18.26	51.4 ± 16.13	47.4 ± 20.35	0.42
	Hemoglobin (g/dL)	12.0 ± 1.75	12.3 ± 1.52	11.7 ± 1.94	0.19
	NT-proBNP (pg/mL)	1302 [498; 2435]	1015 [361; 2251]	1668 [824; 3386]	0.044
	Troponin (pg/mL)	21 [11; 40]	16 [10; 40]	32 [16; 41]	0.47
	CRP (mg/L)	3.1 [1.2; 8.4]	2.1 [1.2; 4.2]	4.7 [1.4; 10.2]	0.045
	Myeloperoxidase (ng/mL)	34.7 [22.7; 44.0]	23.9 [18.4; 32.0]	44.0 [37.8; 78.5]	By design
	Uric acid (mg/dL)	7.3 ± 2.66	6.6 ± 2.34	8.0 ± 2.84	0.06
	Neutrophiles	4.3 ± 1.44	4.3 ± 1.37	4.2 ± 1.54	0.98
	Lymphocytes	1.6 ± 0.66	1.7 ± 0.61	1.5 ± 0.72	0.45
	Monocytes	0.68 ± 0.219	0.65 ± 0.179	0.71 ± 0.257	0.56
	Neutrophile to lymphocyte ratio	3.2 ± 2.12	3.0 ± 1.9	3.3 ± 2.3	0.63
Echocardiography					
	Indexed LA volume (mL/$m^{2}$)	37.6 ± 11.42	36.1 ± 13.29	39.2 ± 9.17	0.32
	LV ejection fraction (%)	57.7 ± 5.11	59.5 ± 4.89	55.8 ± 4.71	0.007
	E wave velocity (mm/s)	108.5 ± 30.29	105.6 ± 23.9	111.4 ± 35.78	0.49
	E/e’ ratio	16.3 ± 5.63	14.4 ± 3.96	18.2 ± 6.40	0.012
	TAPSE (mm)	19.2 ± 6.59	19.9 ± 6.09	18.7 ± 7.17	0.53
	eSPAP (mmHg)	50.7 ± 13.82	49.8 ± 13.32	51.7 ± 14.59	0.66
Vascular function					
	Effective arterial elastance (mmHg/mL)	2.24 ± 0.716	2.43 ± 0.721	2.06 ± 0.676	0.065
	EndoPAT	(n = 45)	(n = 22)	(n = 23)	0.55
	Reactive hyperemia index (RHI)	1.67 [1.33; 2.02]	1.66 [1.32; 1.95]	1.82 [1.34; 2.30]	
	Augmentation Index (AIx)	17.81 [2.64; 31.24]	19.9 [10.5; 33.4]	11.1 [0.1; 30.7]	0.018

NYHA, New York heart association; COPD, chronic obstructive pulmonary disease; MRA, mineralocorticoid receptor antagonist; ACE, angiotensin-converting enzyme; ARB, angiotensin II receptor blocker; eGRF, estimated glomerular filtration rate; NT-proBNP, N-terminal of brain natriuretic peptide; CRP, C-reactive protein; LA, left atrium; LV, left ventricle; TAPSE, tricuspid annular plane systolic excursion; eSPAP, estimated systolic pulmonary artery pressure. *p* values are for differences of characteristics between the groups MPO above median vs MPO below median and are derived from independent sample *t*-test, Mann Whitney U test, Chi-square test or Fisher exact test when appropriate). All tests were 2-sided and *p*-value < 0.05 was considered statistically significant.

**Fig. 2.**
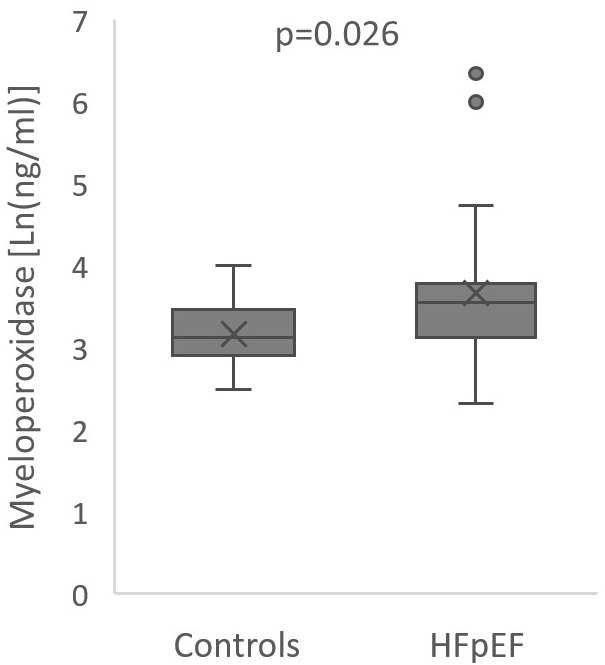
**Boxplot of myeloperoxidase levels in heart failure and preserved ejection fraction patients and controls**. Center line: median; box limits: upper and lower quartiles; whiskers: 1.5 × interquartile range; cross: mean; points: outliers.

Besides expected differences in NT-proBNP levels and echocardiographic parameters, patients with HFpEF had higher levels of CRP (3.1 mg/L [1.2; 8.4] vs 1.2 mg/L [1.0; 1.75], *p* = 0.001), uric acid (7.3 ± 2.66 vs 5.2 ± 1.01, *p *< 0.001) and MPO (34.7 ng/mL [22.7; 44.0] vs 22.6 [18.2; 32.0], *p* = 0.026) reflecting higher degree of inflammation and oxidative stress (Fig. 2). However, there were no significant differences in vascular function. In controls, the reactive hyperemia index was 1.80 [1.42; 2.55] and the augmentation index 17.7 [4.6; 36.9] vs 1.67 [1.33; 2.02] and 17.81 [2.64; 31.24] in patients (respectively *p* = 0.26 and 0.70). Effective arterial elastance was also not different (1.99 ± 0.570 vs 2.24 ± 0.716, *p* = 0.21).

The AUC of the ROC curves for myeloperoxidase was 0.72 (0.59; 0.84) *p* = 0.006 indicating moderate diagnostic value for HFpEF. Expectedly, NT-proBNP levels had a very good diagnostic value of 0.94 (0.89; 1.00) *p *< 0.001 (**Supplementary Fig. 1**).

### 3.2 Correlation between MPO Levels and Patients’ Characteristics 

**Fig. 3.**
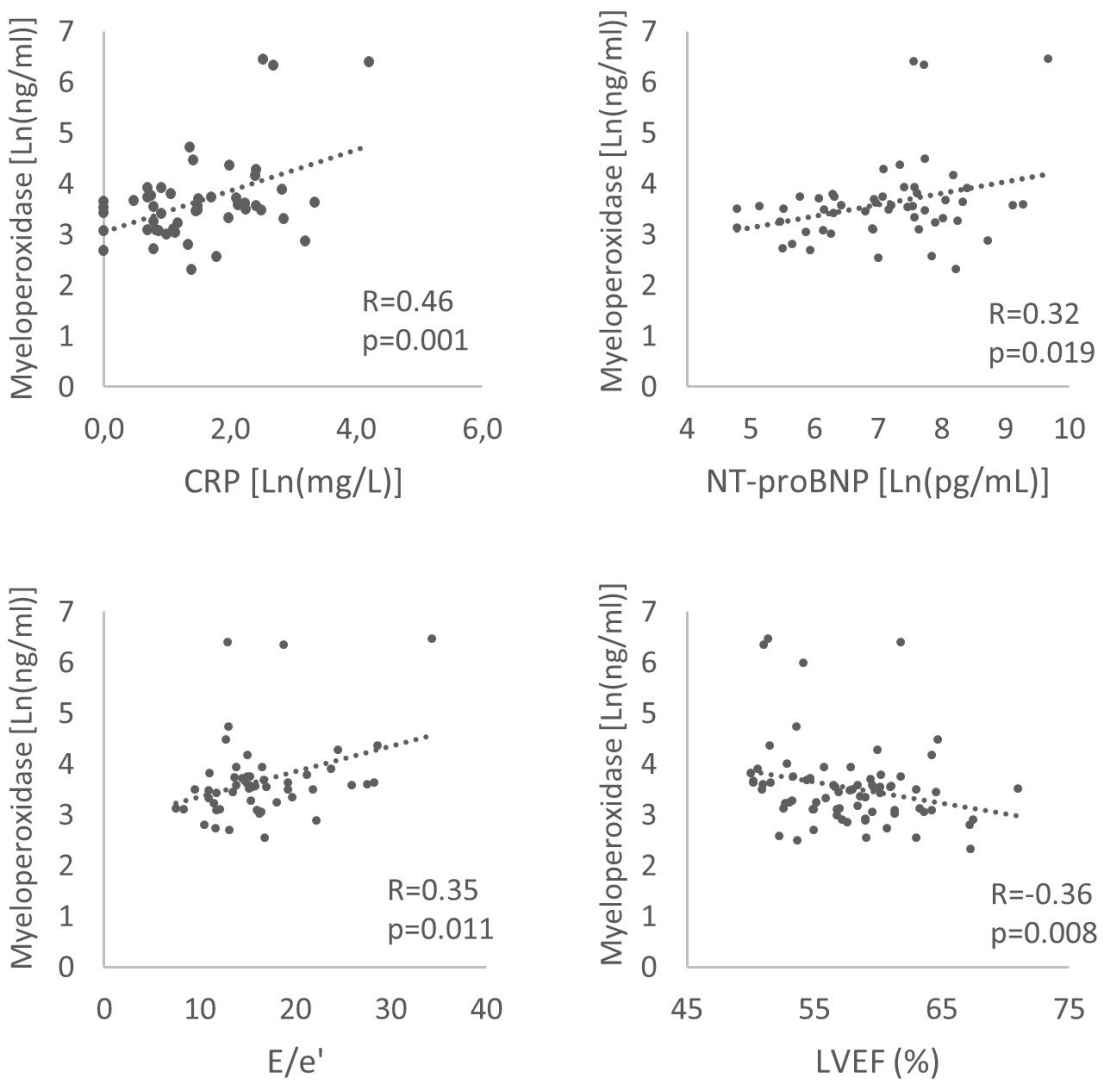
**Correlations between myeloperoxidase and C-reactive protein (CRP), NT-proBNP, E/e’ ratio, left ventricular ejection fraction (LVEF) in heart failure and preserved ejection fraction patients**.

Among HFpEF patients, MPO levels were correlated with markers of inflammation; CRP (R = 0.46, *p* = 0.001) and neutrophile to lymphocyte ratio (R = 0.36, *p* = 0.031) and with signs of LV remodelling and elevated filling pressures, namely NT-proBNP levels (R = 0.32, *p* = 0.019), decreased LV ejection fraction (LVEF, R = –0.36, *p* = 0.008) and E/e’ ratio (R = 0.35, *p* = 0.011) (Fig. 3). There was no correlation with age (R = 0.12, *p* = 0.41), body mass index (R = 0.09, *p* = 0.54), nor renal function (glomerular filtration rate estimated by Chronic Kidney Disease Epidemiology Collaboration CKD-EPI equation) (R = –0.13, *p* = 0.34) [14].

### 3.3 Characteristics Associated with High MPO Levels 

Patients with MPO levels above the median consistently had higher levels of CRP and NT-proBNP levels. They also showed lower LVEF (55.8 ± 4.71% vs 59.5 ± 4.89%, *p* = 0.007) and higher E/e’ ratio (18.2 ± 6.40 vs 14.4 ± 3.96, *p* = 0.012). Patients with MPO levels above the median suffered more often from diabetes (48 vs 18%, *p* = 0.017) and were more often males (48 vs 21%, *p* = 0.037) than patients with MPO levels below the median (Table 1). Prevalence of other risk factors (hypertension, hypercholesterolemia) and respiratory comorbidities (chronic obstructive pulmonary disease (COPD) and sleep apneas) did not differ between groups.

**Fig. 4.**
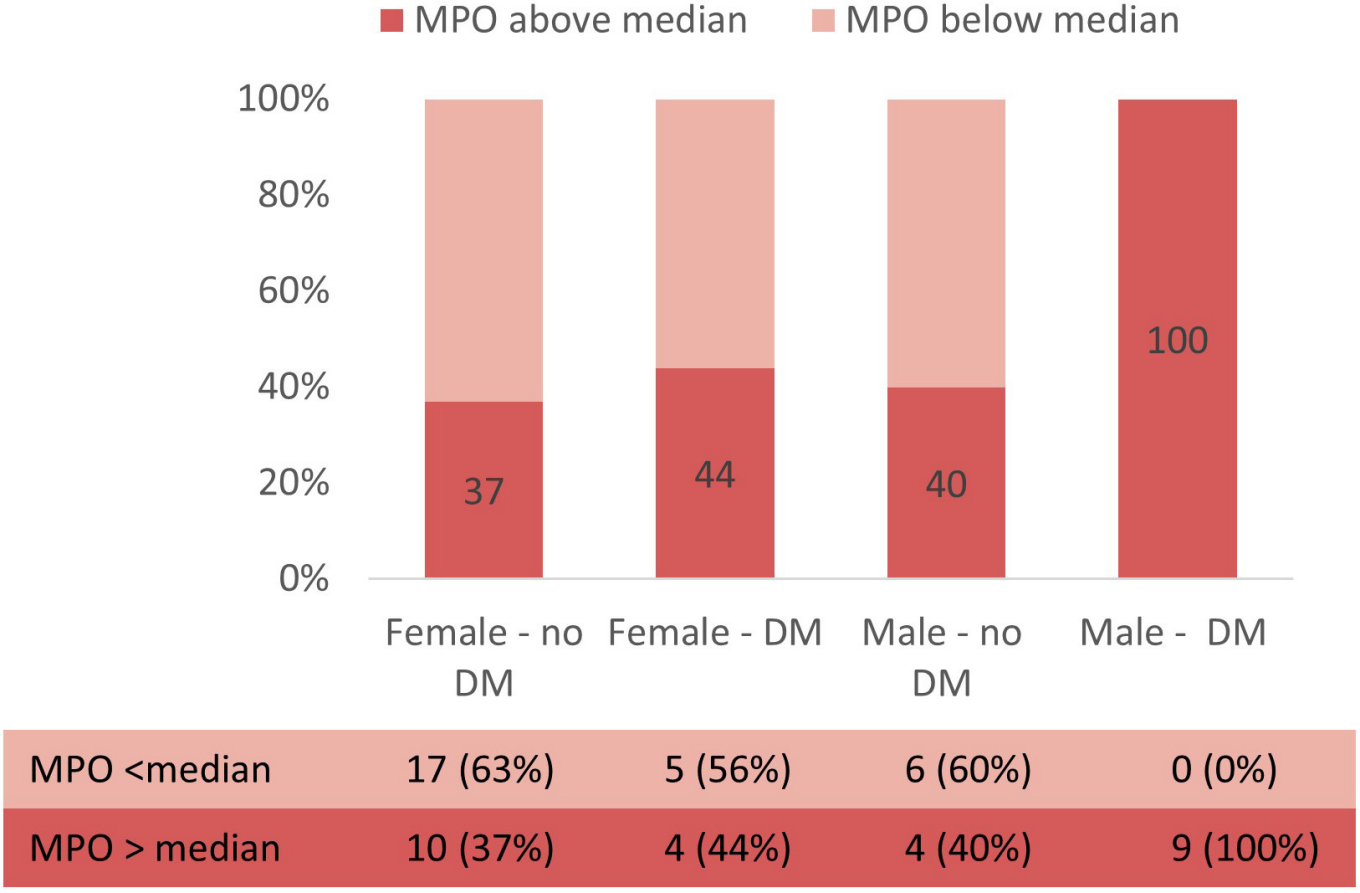
**Proportion of patients with MPO levels above or below median according to sex and diabetic status**.

In multivariable logistic regression, diabetic status remained predictive of high levels of myeloperoxidase after adjustment for age and sex (OR = 4.7, 95% CI 1.15–19.19, *p* = 0.031). Fig. 4 illustrates the proportion of patients with MPO levels above or below median according to sex and diabetic status. Interestingly, all men suffering from diabetes (9, 100%) had MPO levels above the median, while in women (both with or without diabetes) and in men without diabetes the proportion was similar, around 40%.

Intriguingly, patients with higher levels of MPO showed lower augmentation index (11.1 [0.1; 30.7] vs 19.9 [10.5; 33.4], *p* = 0.018) and a trend towards lower effective arterial elastance (2.06 ± 0.676 vs 2.43 ± 0.721, *p* = 0.065) indicating less vascular stiffness. Endothelial function did not differ between groups (1.82 [1.34; 2.30] vs 1.66 [1.32; 1.95], *p* = 0.55).

## 4. Discussion 

The findings of this study are as follows: patients with HFpEF have higher levels of MPO than controls, MPO levels in HFpEF are positively correlated with inflammation (CRP levels), diastolic dysfunction (E/e’) and congestion (NT-proBNP) and negatively with left ventricular ejection fraction. Patients with MPO levels above the median suffer more often from diabetes, are more often males but tend to show less vascular stiffness (lower AIx) than patients with MPO levels below the median.

Several studies have shown a strong correlation between MPO and cardiovascular disease (CVD) including acute coronary syndrome, atherosclerosis, hypertension, and stroke [4, 15]. Consistently, recent studies that target MPO in animal models of CVD have demonstrated favourable outcomes with regard to disease progression [16]. However, data in HFpEF are limited to the study by Hage and collegues [6]. Our study corroborates their finding that MPO is elevated in HFpEF patients compared to controls and demonstrates that this applies also when the control group is older (74 ± 6 years) and with a proportion of women comparable to the HFpEF group (65 and 72% respectively). MPO levels showed moderate diagnostic value for HFpEF, less powerful in that regard than NT-proBNP levels (ROC curves **Supplementary Fig. 1**).

MPO-mediated oxidative stress may be one of the mechanistic link between comorbidities, inflammation and endothelial dysfunction at the source of HFpEF [3]. Comorbidities, namely obesity, diabetes, and ageing generate inflammation [17, 18, 19, 20], during which MPO is released and uses $H_{2}$$O_{2}$ as a substrate to produce hypochlorous acid, a potent pro-oxidant and proinflammatory molecule. MPO levels in our study were indeed correlated with markers of inflammation (CRP and NLR) and signs of myocardial remodelling, namely NT-proBNP levels, decreasing LVEF (although within the normal range) and increasing E/e’. It is still to be determined whether MPO plays a causative role in the development of the disease or if it is merely a bystander of neutrophils activation. Indeed, recent studies directly incriminate activated neutrophils in aggravating diastolic dysfunction in mice subject to pressure overload [21], and in HFpEF patients [22, 23].

High MPO levels were associated with diabetic status. This is not surprising since diabetes is known to promote a systemic pro-inflammatory state [24, 25]. Furthermore, MPO was shown to be predictive of insulin resistance in a population of obese patients [26]. Since the proportion of patients with diabetes was higher in patients with MPO levels above the median, and since the difference in pathophysiology between diabetic and non-diabetic HFpEF patients is a topic of interest [27, 28], we further investigated this association with logistic regression adjusted for age and sex. Interestingly, the combination of male sex and diabetic status seem particularly associated with higher levels of MPO among patients with HFpEF. Indeed, all men suffering from diabetes had MPO levels above the median, while the proportion was limited to 40% in the other subgroups (Fig. 4). This finding is consistent with the sex-specific proteomic profile of patients with HFpEF in the PROMIS study [29], where they demonstrated that inflammation-related pathways predominated in men.

**Fig. 5.**
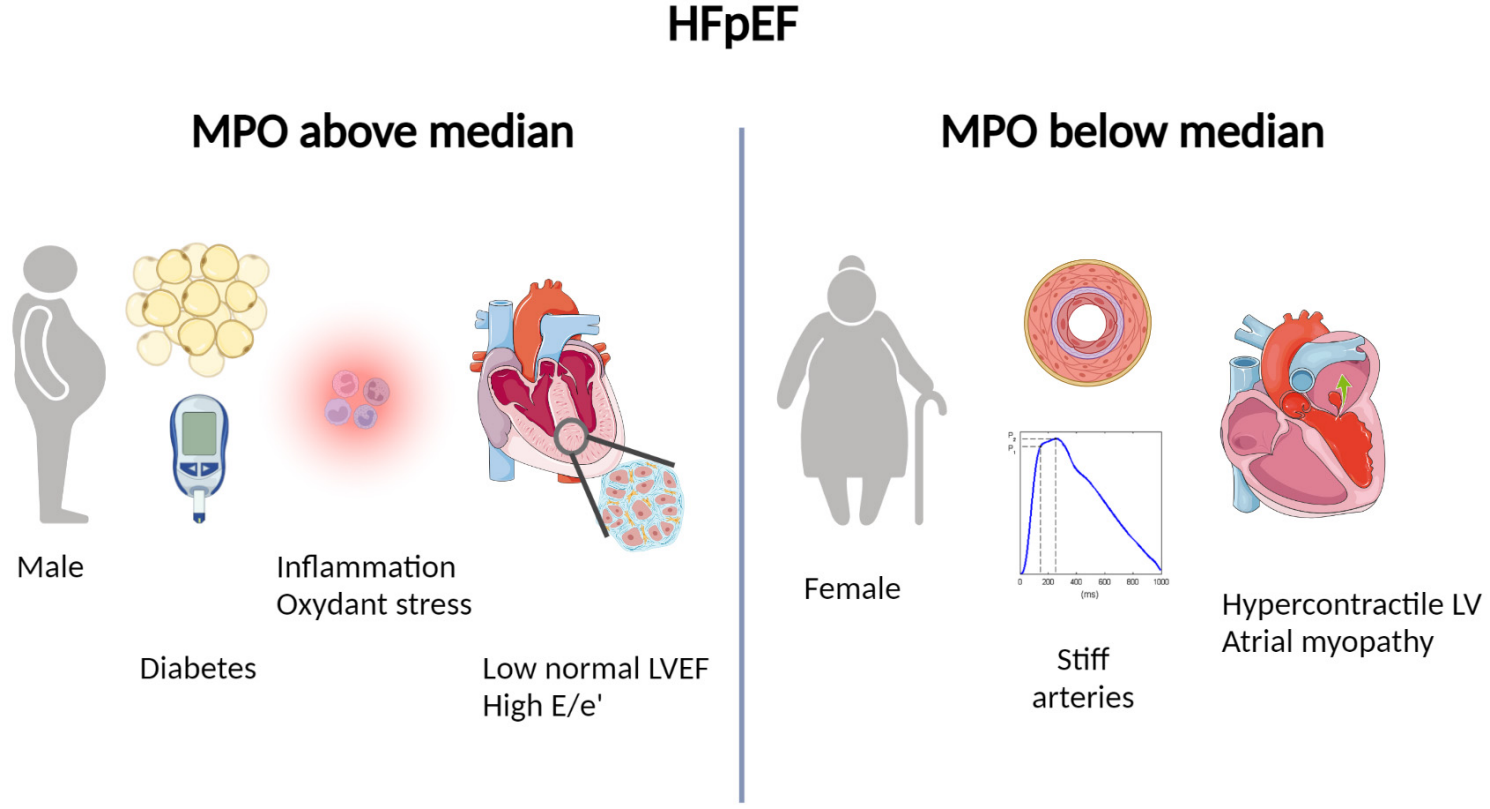
**Illustration of patients’ characteristics associated with levels of myeloperoxidase below or above the median**. Patients with heart failure and preserved ejection fraction and myeloperoxidase above the median are more often men, suffer more often form diabetes, show subtle left ventricular dysfunction and pronounced diastolic dysfunction (high E/e’) while patients with myeloperoxidase below the median are more often women with elevated vascular stiffness and high left ventricular ejection fraction.

On the other hand, we found no association between vascular stiffness or endothelial function and MPO levels. Even more surprising, vascular stiffness seemed less important in the patients with higher MPO levels (lower AIx, lower Ea). The augmentation index (AIx) is calculated from pulse waveforms as the ratio of the difference between the early and late systolic peaks of the waveform relative to the early peak (Fig. 1) and represents the relative importance of the reflected wave [30]. Multiple small reflections travel back to the proximal aorta and merge into a “net” reflected wave whose magnitude and timing depend on vascular stiffness. In older subjects, systolic wave reflections mediate late systolic load, with an important impact on LV relaxation [31, 32]. The augmentation index is not simply a measure of arterial stiffness and wave reflection, but was also shown to be elevated in conditions of increased LV contractility and may reflect overall ventricular-vascular coupling [33]. In HFpEF, high AIx was associated with abnormal LV diastolic responses to exercise, particularly in women, suggesting that arterial stiffness may contribute to the pathophysiology of HFpEF more commonly in women than in men [34]. The finding that patients with MPO levels above median do not display more endothelial dysfunction, nor vascular stiffness might be an indication that the sequence: comorbidities, inflammation, oxidative stress, endothelial dysfunction, myocardial remodelling is not straightforward. Rather, different mechanisms are probably involved in the development of myocardial remodeling and impaired vascular function, while both condition can ultimately lead to HFpEF. Recent data from phenomapping point towards the same direction. Indeed, although studies identify slightly different clusters depending on available variables [35, 36, 37, 38, 39], two clusters seem to be commonly differentiated: one with older patients with stiff arteries, small highly contractile LVs and high rates of electrical remodelling (atrial fibrillation) and the other with high rates of metabolic comorbidities, mainly diabetes, marked LV remodelling and advanced diastolic dysfunction. Inflammation and oxidative stress may play a more prominent role in the latter, hence the elevation of MPO (Fig. 5). Accordingly, there were more men and more patients suffering from diabetes in the group of patients with MPO levels above the median and they displayed lower (although ≥50%) LVEF and higher E/e’. These two subgroups might reflect two distinct pathophysiological mechanisms underlying HFpEF.

## 5. Strengths and Limitations

We acknowledge this single centre study has several limitations. Maybe the most important arising from the small sample size. Unfortunately, restrictions related to the COVID pandemic interrupted the recruitment for several months. Furthermore, due to limitations of the EndoPAT technique, we could not obtain RHI and AIx for all patients. Despite our best effort to include controls of similar age and sex, both groups are not accurately matched for these characteristics. However, our groups are more alike than the only other published study demonstrating higher MPO levels in HFpEF [6]. The presence of a control group of similar age and sex is important since there are no validated reference values of MPO. On the contrary, there is a wide range of concentrations reported in the literature [40, 41, 42], hence standardisation will be necessary before routine use of MPO measurements.

In the context of the development of treatment with MPO inhibitor “AZD4831” (NCT03611153) it is interesting to note that not all patients might respond homogeneously. The results of our study suggest that patients with metabolic comorbidities, particularly diabetes, subtle LV dysfunction and evident diastolic dysfunction might benefit more from treatment targeting MPO while patients with predominant arterial stiffness (mostly females) and hyper contractile LV might be less responsive. Hence, while this study should be considered exploratory and hypothesis generating, it adds relevant information to existing literature. Future studies should aim at exploring the sex specific interplay between vascular inflammation and stiffness in this population, with special interest in features of metabolic stress such as obesity and diabetes.

## 6. Conclusions

Myeloperoxidase levels are elevated in HFpEF compared to controls, reflecting leukocyte activation and oxidative stress. Patients with levels of MPO above the median are more often males and suffer more often from diabetes. MPO levels in HFpEF are positively correlated with diastolic dysfunction and congestion and negatively with left ventricular ejection fraction. The association between oxidative stress and vascular stiffness, on the other hand could not be demonstrated and deserves future attention.

## Data Availability

Data available on reasonable request.

## Supplementary Material

Supplementary material associated with this article can be found, in the online version, at https://doi.org/10.31083/j.rcm2402056.

## References

[1] Lam CSP, Voors AA, de Boer RA, Solomon SD, van Veldhuisen DJ (2018). Heart failure with preserved ejection fraction: from mechanisms to therapies. *European Heart Journal*.

[2] Paulus WJ, Tschöpe C (2013). A novel paradigm for heart failure with preserved ejection fraction: comorbidities drive myocardial dysfunction and remodeling through coronary microvascular endothelial inflammation. *Journal of the American College of Cardiology*.

[3] Sousa T, Reina-Couto M, Gomes P, Chakraborti S, Dhalla N, Ganguly N, Dikshit M (2019). Role of Oxidative Stress in the Pathophysiology of Arterial Hypertension and Heart Failure. *Oxidative Stress in Heart Diseases*.

[4] Tang WHW, Tong W, Troughton RW, Martin MG, Shrestha K, Borowski A (2007). Prognostic value and echocardiographic determinants of plasma myeloperoxidase levels in chronic heart failure. *Journal of the American College of Cardiology*.

[5] Rudolph V, Andrié RP, Rudolph TK, Friedrichs K, Klinke A, Hirsch-Hoffmann B (2010). Myeloperoxidase acts as a profibrotic mediator of atrial fibrillation. *Nature Medicine*.

[6] Hage C, Michaëlsson E, Kull B, Miliotis T, Svedlund S, Linde C (2020). Myeloperoxidase and related biomarkers are suggestive footprints of endothelial microvascular inflammation in HFpEF patients. *ESC Heart Failure*.

[7] Ponikowski P, Voors AA, Anker SD, Bueno H, Cleland JGF, Coats AJS (2016). 2016 ESC Guidelines for the diagnosis and treatment of acute and chronic heart failure: The Task Force for the diagnosis and treatment of acute and chronic heart failure of the European Society of Cardiology (ESC). Developed with the special contribution of the Heart Failure Association (HFA) of the ESC. *European Journal of Heart Failure*.

[8] McDonagh TA, Metra M, Adamo M, Gardner RS, Baumbach A, Böhm M (2021). 2021 ESC Guidelines for the diagnosis and treatment of acute and chronic heart failure. *European Heart Journal*.

[9] Mitchell C, Rahko PS, Blauwet LA, Canaday B, Finstuen JA, Foster MC (2019). Guidelines for Performing a Comprehensive Transthoracic Echocardiographic Examination in Adults: Recommendations from the American Society of Echocardiography. *Journal of the American Society of Echocardiography*.

[10] Nagueh SF, Smiseth OA, Appleton CP, Byrd BF, Dokainish H, Edvardsen T (2016). Recommendations for the Evaluation of Left Ventricular Diastolic Function by Echocardiography: An Update from the American Society of Echocardiography and the European Association of Cardiovascular Imaging. *European Heart Journal-Cardiovascular Imaging*.

[11] Lam CSP, Roger VL, Rodeheffer RJ, Bursi F, Borlaug BA, Ommen SR (2007). Cardiac structure and ventricular-vascular function in persons with heart failure and preserved ejection fraction from Olmsted County, Minnesota. *Circulation*.

[12] Kelly RP, Ting CT, Yang TM, Liu CP, Maughan WL, Chang MS (1992). Effective arterial elastance as index of arterial vascular load in humans. *Circulation*.

[13] Perrault R, Omelchenko A, Taylor CG, Zahradka P (2019). Establishing the interchangeability of arterial stiffness but not endothelial function parameters in healthy individuals. *BMC Cardiovascular Disorders*.

[14] Levey AS, Stevens LA, Schmid CH, Zhang YL, Castro AF, Feldman HI (2009). A new equation to estimate glomerular filtration rate. *Annals of Internal Medicine*.

[15] Nicholls SJ, Hazen SL (2005). Myeloperoxidase and cardiovascular disease. *Arteriosclerosis, Thrombosis, and Vascular Biology*.

[16] Ramachandra CJA, Ja KPMM, Chua J, Cong S, Shim W, Hausenloy DJ (2020). Myeloperoxidase as a Multifaceted Target for Cardiovascular Protection. *Antioxidants & Redox Signaling*.

[17] Franssen C, Chen S, Unger A, Korkmaz HI, De Keulenaer GW, Tschöpe C (2016). Myocardial Microvascular Inflammatory Endothelial Activation in Heart Failure with Preserved Ejection Fraction. *JACC: Heart Failure*.

[18] Kolijn D, Pabel S, Tian Y, Lódi M, Herwig M, Carrizzo A (2021). Empagliflozin improves endothelial and cardiomyocyte function in human heart failure with preserved ejection fraction via reduced pro-inflammatory-oxidative pathways and protein kinase Gα oxidation. *Cardiovascular Research*.

[19] Giacco F, Brownlee M (2010). Oxidative stress and diabetic complications. *Circulation Research*.

[20] Rizvi F, Preston CC, Emelyanova L, Yousufuddin M, Viqar M, Dakwar O (2021). Effects of Aging on Cardiac Oxidative Stress and Transcriptional Changes in Pathways of Reactive Oxygen Species Generation and Clearance. *Journal of the American Heart Association*.

[21] Wang Y, Sano S, Oshima K, Sano M, Watanabe Y, Katanasaka Y (2019). Wnt5a-Mediated Neutrophil Recruitment Has an Obligatory Role in Pressure Overload-Induced Cardiac Dysfunction. *Circulation*.

[22] Bai B, Cheng M, Jiang L, Xu J, Chen H, Xu Y (2021). High Neutrophil to Lymphocyte Ratio and Its Gene Signatures Correlate with Diastolic Dysfunction in Heart Failure with Preserved Ejection Fraction. *Frontiers in Cardiovascular Medicine*.

[23] Boralkar KA, Kobayashi Y, Amsallem M, Ataam JA, Moneghetti KJ, Cauwenberghs N (2020). Value of Neutrophil to Lymphocyte Ratio and Its Trajectory in Patients Hospitalized with Acute Heart Failure and Preserved Ejection Fraction. *The American Journal of Cardiology*.

[24] Ritchie RH, Abel ED (2020). Basic Mechanisms of Diabetic Heart Disease. *Circulation Research*.

[25] Tromp J, Voors AA, Sharma A, Ferreira JP, Ouwerkerk W, Hillege HL (2020). Distinct Pathological Pathways in Patients with Heart Failure and Diabetes. *JACC: Heart Failure*.

[26] Gómez García A, Rivera Rodríguez M, Gómez Alonso C, Rodríguez Ochoa DY, Alvarez Aguilar C (2015). Myeloperoxidase is associated with insulin resistance and inflammation in overweight subjects with first-degree relatives with type 2 diabetes mellitus. *Diabetes & Metabolism Journal*.

[27] Lejeune S, Roy C, Slimani A, Pasquet A, Vancraeynest D, Vanoverschelde J (2021). Diabetic phenotype and prognosis of patients with heart failure and preserved ejection fraction in a real life cohort. *Cardiovascular Diabetology*.

[28] Hulot J, Livrozet M (2021). HFpEF: Should We Consider Diabetic Patients Separately? The Cardiomyocytes Say Yes. *Journal of the American College of Cardiology*.

[29] Chandramouli C, Ting TW, Tromp J, Agarwal A, Svedlund S, Saraste A (2022). Sex differences in proteomic correlates of coronary microvascular dysfunction among patients with heart failure and preserved ejection fraction. *European Journal of Heart Failure*.

[30] Borlaug BA, Melenovsky V, Redfield MM, Kessler K, Chang H, Abraham TP (2007). Impact of arterial load and loading sequence on left ventricular tissue velocities in humans. *Journal of the American College of Cardiology*.

[31] Yano M, Kohno M, Kobayashi S, Obayashi M, Seki K, Ohkusa T (2001). Influence of timing and magnitude of arterial wave reflection on left ventricular relaxation. *American Journal of Physiology. Heart and Circulatory Physiology*.

[32] Kawaguchi M, Hay I, Fetics B, Kass DA (2003). Combined ventricular systolic and arterial stiffening in patients with heart failure and preserved ejection fraction: implications for systolic and diastolic reserve limitations. *Circulation*.

[33] Heffernan KS, Patvardhan EA, Hession M, Ruan J, Karas RH, Kuvin JT (2010). Elevated augmentation index derived from peripheral arterial tonometry is associated with abnormal ventricular-vascular coupling. *Clinical Physiology and Functional Imaging*.

[34] Lau ES, Panah LG, Zern EK, Liu EE, Farrell R, Schoenike MW (2022). Arterial Stiffness and Vascular Load in HFpEF: Differences Among Women and Men. *Journal of Cardiac Failure*.

[35] Cohen JB, Schrauben SJ, Zhao L, Basso MD, Cvijic ME, Li Z (2020). Clinical Phenogroups in Heart Failure with Preserved Ejection Fraction: Detailed Phenotypes, Prognosis, and Response to Spironolactone. *JACC: Heart Failure*.

[36] Shah SJ, Katz DH, Selvaraj S, Burke MA, Yancy CW, Gheorghiade M (2015). Phenomapping for novel classification of heart failure with preserved ejection fraction. *Circulation*.

[37] Hedman ÅK, Hage C, Sharma A, Brosnan MJ, Buckbinder L, Gan L (2020). Identification of novel pheno-groups in heart failure with preserved ejection fraction using machine learning. *Heart*.

[38] Kao DP, Lewsey JD, Anand IS, Massie BM, Zile MR, Carson PE (2015). Characterization of subgroups of heart failure patients with preserved ejection fraction with possible implications for prognosis and treatment response. *European Journal of Heart Failure*.

[39] Galli E, Bourg C, Kosmala W, Oger E, Donal E (2021). Phenomapping Heart Failure with Preserved Ejection Fraction Using Machine Learning Cluster Analysis: Prognostic and Therapeutic Implications. *Heart Failure Clinics*.

[40] Gar C, Thorand B, Herder C, Sujana C, Heier M, Meisinger C (2022). Association of circulating MR-proADM with all-cause and cardiovascular mortality in the general population: Results from the KORA F4 cohort study. *PLoS ONE*.

[41] Zsíros N, Koncsos P, Lőrincz H, Seres I, Katkó M, Szentpéteri A (2016). Paraoxonase-1 arylesterase activity is an independent predictor of myeloperoxidase levels in overweight patients with or without cardiovascular complications. *Clinical Biochemistry*.

[42] Luetkens JA, Wolpers AC, Beiert T, Kuetting D, Dabir D, Homsi R (2018). Cardiac magnetic resonance using late gadolinium enhancement and atrial T1 mapping predicts poor outcome in patients with atrial fibrillation after catheter ablation therapy. *Scientific Reports*.

