# Supplementary material for: Association of Plasma Myeloperoxidase with Inflammation and Diabetic status in HFpEF
Source: Rev Cardiovasc Med. 2023 Feb 8;24(2):56. doi: 10.31083/j.rcm2402056 (PMC11273114; doi:10.31083/j.rcm2402056)
Supplement: Supplementary file 1 [file 2153-8174-24-2-056-s1.zip › 2153-8174-24-2-056-s1.docx]

Supplementary material

| **Supplementary Table 1. Baseline characteristics of the control group.** | | |
| --- | --- | --- |
|  | Controls  N=18 | Patients  N=55 |
| Age (years) | 75 ± 5.0 | 80 ± 8.7 |
| Female (n, %) | 13 (72%) | 36 (65%) |
| Body mass index (kg/m^2^) | 24 ± 2.8 | 28 ± 5.0 |
| Systolic blood pressure (mmHg) | 125 ± 15 | 134 ± 20 |
| Diastolic blood pressure (mmHg) | 77 ± 9 | 74 ± 14 |
| Heart rate at inclusion (bpm) | 63 ± 9 | 72 ± 13 |
| NYHA III – IV (n, %) | 0 (0%) | 20 (36%) |
| Diabetes (n,%) | 1 (6%) | 18 (33%) |
| Smoking (n, %) | 2 (11%) | 18 (18%) |
| Hypertension (n, %) | 13 (72%) | 52 (95%) |
| Hypercholesterolemia (n, %) | 10 (56%) | 39 (71%) |
| Sleep apneas (n, %) | 0 (0%) | 6 (11%) |
| COPD (n, %) | 0 (0%) | 6 (11%) |
| Loopdiuretics (n, %) | 0 (0%) | 42 (76%) |
| MRA (n, %) | 0 (0%) | 18 (33%) |
| Beta blockers (n, %) | 2 (11%) | 34 (62%) |
| ACE inhibitors/ARB (n, %) | 6 (33%) | 43 (78%) |
| Statins (n,%) | 1 (6%) | 35 (65%) |
| eGFR (ml/min/1.73m^2^) | 70 ± 15.6 | 49 ± 18.3 |
| Hemoglobin (g/dL) | 13 ± 0.9 | 12 ± 1.8 |
| NT-proBNP (pg/mL) | 128 [90 ; 236] | 1302 [498 ; 2435] |
| Troponin (pg/mL) | 8 [5 ; 11] | 21 [11 ; 40] |
| CRP (mg/L) | 1.2 [1.0 ; 1.75] | 3.1 [1.2 ; 8.4] |
| Myeloperoxidase (ng/ml) | 22.6 [18.2 ; 32.0] | 34.7 [22.7 ; 44.0] |
| Uric acid (mg/dL) | 5.2 ± 1.01 | 7.3 ± 2.66 |
| Neutrophiles (10^3^/µL) | 3.7 ± 1.29 | 4.3 ± 1.44 |
| Lymphocytes (10^3^/µL) | 1.7 ± 0.51 | 1.6 ± 0.66 |
| Monocytes (10^3^/µL) | 0.57 ± 0.222 | 0.68 ± 0.219 |
| Neutrophile to lymphocyte ratio | 2.4 ± 1.14 | 3.2 ± 2.12 |
| Indexed LA volume (mL/m^2^) | 21.9 ± 9.40 | 37.6 ± 11.42 |
| LV ejection fraction (%) | 57.7 ± 3.86 | 57.7 ± 5.11 |
| E/e’ ratio | 9.8 ± 2.64 | 16.3 ± 5.63 |
| Effective arterial elastance (mmHg/mL) | 1.99 ± 0.570 | 2.24 ± 0.716 |
| EndoPAT  Reactive hyperemia index (RHI) | 1.80 [1.42 ; 2.55] | 1.67 [1.33 ; 2.02] |
| Augmentation Index (AIx) | 17.7 [4.6 ; 36.9] | 17.81 [2.64 ; 31.24] |
| NYHA: New York heart association, COPD: chronic obstructive pulmonary disease, MRA: mineralocorticoid receptor antagonist, ACE: angiotensin-converting enzyme, ARB: angiotensin II receptor blocker, eGRF: estimated glomerular filtration rate, NT-proBNP: N-terminal of brain natriuretic peptide, CRP: C-reactive protein, LA: left atrium, LV: left ventricle | | |

**Supplementary Figure 1. Receiver operating characteristic curves of plasma myeloperoxidase levels and NT-proBNP levels to diagnose HFpEF.**


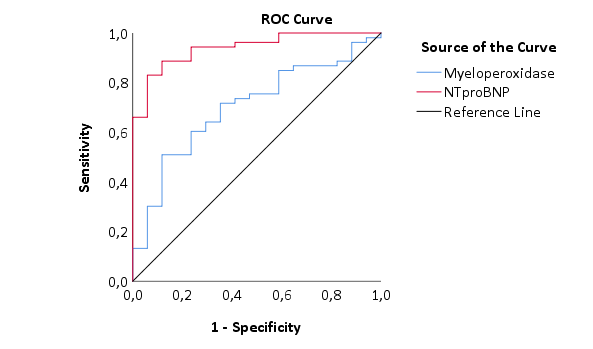


**Area under the curve**Myeloperoxidase: 0.72 (0.59 ; 0.84) p=0.006
NT-proBNP: 0.94 (0.89 ; 1.00) p<0.001
